# Acute Liver Injury Is Independent of B Cells or Immunoglobulin M

**DOI:** 10.1371/journal.pone.0138688

**Published:** 2015-09-25

**Authors:** James A. Richards, Martina Bucsaiova, Emily E. Hesketh, Chiara Ventre, Neil C. Henderson, Kenneth Simpson, Christopher O. C. Bellamy, Sarah E. M. Howie, Stephen M. Anderton, Jeremy Hughes, Stephen J. Wigmore

**Affiliations:** 1 MRC Centre for Inflammation Research, The University of Edinburgh, Edinburgh, United Kingdom; 2 Clinical Surgery, The University of Edinburgh, Edinburgh, United Kingdom; 3 Hepatology, Division of Health Sciences, School of Clinical Sciences, University of Edinburgh, Edinburgh, United Kingdom; 4 The University of Edinburgh, Edinburgh, United Kingdom; 5 Department of Pathology, Royal Infirmary of Edinburgh, Edinburgh, United Kingdom; University of Leicester, UNITED KINGDOM

## Abstract

**Background & Aims:**

Acute liver injury is a clinically important pathology and results in the release of Danger Associated Molecular Patterns, which initiate an immune response. Withdrawal of the injurious agent and curtailing any pathogenic secondary immune response may allow spontaneous resolution of injury. The role B cells and Immunoglobulin M (IgM) play in acute liver injury is largely unknown and it was proposed that B cells and/or IgM would play a significant role in its pathogenesis.

**Methods:**

Tissue from 3 models of experimental liver injury (ischemia-reperfusion injury, concanavalin A hepatitis and paracetamol-induced liver injury) and patients transplanted following paracetamol overdose were stained for evidence of IgM deposition. Mice deficient in B cells (and IgM) were used to dissect out the role B cells and/or IgM played in the development or resolution of injury. Serum transfer into mice lacking IgM was used to establish the role IgM plays in injury.

**Results:**

Significant deposition of IgM was seen in the explanted livers of patients transplanted following paracetamol overdose as well as in 3 experimental models of acute liver injury (ischemia-reperfusion injury, concanavalin A hepatitis and paracetamol-induced liver injury). Serum transfer into IgM-deficient mice failed to reconstitute injury (p = 0.66), despite successful engraftment of IgM. Mice deficient in both T and B cells (RAG1-/-) mice (p<0.001), but not B cell deficient (μMT) mice (p = 0.93), were significantly protected from injury. Further interrogation with T cell deficient (CD3εKO) mice confirmed that the T cell component is a key mediator of sterile liver injury. Mice deficient in B cells and IgM mice did not have a significant delay in resolution following acute liver injury.

**Discussion:**

IgM deposition appears to be common feature of both human and murine sterile liver injury. However, neither IgM nor B cells, play a significant role in the development of or resolution from acute liver injury. T cells appear to be key mediators of injury. In conclusion, the therapeutic targeting of IgM or B cells (e.g. with Rituximab) would have limited benefit in protecting patients from acute liver injury.

## Background

The term acute liver injury (ALI) encompasses a spectrum of sterile or infective hepatocellular insults characterised by acute inflammation within the liver. Injury results in the release of Danger Associated Molecular Patterns (DAMPs), which initiate an immune response. Withdrawal of the injurious agent and curtailing any pathogenic secondary immune response may allow spontaneous resolution of injury [1, 2].

ALI may progress to acute liver failure, which is associated with a mortality of up to 50% [3, 4]. In the developing world, infections (esp. Hepatitis A, B and E viruses) are the commonest aetiology, whereas in the developed world sterile causes predominate [3, 5]. Sterile triggers include drug toxicity (mainly paracetamol/acetaminophen toxicity), autoimmunity and ischemia (ischemia-reperfusion injury (IRI), hypoxic hepatitis). Survival is improving as a result of early diagnosis, improvements in critical care and the growing use of emergency liver transplantation [6]. However, there is still an unmet clinical need to understand how intervention targeting the secondary immune response can benefit patients at risk, or in the early phases, of ALI. One such scenario is ischaemia-reperfusion injury during liver resection or transplantation.

IRI results from the interruption then reinstatement of an organ’s blood supply. It limits access to donor organs and has been linked to early graft failure, as well as both acute and chronic rejection [7, 8]. IRI involves both ischemic and immune-mediated reperfusion phases of injury; numerous mediators and immune cells have been identified as being important in the evolution of this injury and common pathways appear to exist in the pathogenesis of IRI irrespective of the affected organ [9, 10]. Early elevation in pro-inflammatory cytokines in patients following liver resection surgery is linked to worse clinical outcome [11].

B cells are capable of shaping the nature of an immune response through their ability to present antigen and via their ability to produce both cytokines and antibodies. This may have a pro-inflammatory or regulatory influence on the resulting immune response [12]. B cells have been shown to have a pathogenic role in anti-CD40-induced liver injury [13] and in fibrotic liver disease [14]. Numata and colleagues have previously published that mice deficient in both B and T cells (RAG2-/-) had significantly reduced injury compared to wildtype controls 6 hours following administration of a toxic dose of paracetamol [15]. Similarly, mice deficient in both B and T cells (RAG1-/-) were also protected from hepatic IRI 24 hours post-operatively [16, 17]. B cells have a pathogenic role in the aetiology of renal IRI, with mice deficient in B cells (μMT) being protected injury [18]. The presence of B cells in the post-ischemic kidney was also associated with lower levels of IL-10 and slower resolution of injury [19].

Immunoglobulin M (IgM) is produced by B cells and is a normal component of serum. Natural IgM is thought to be produced as a response to self-antigen; it is polyreactive and has a role in the clearance of apoptotic cells [20]. IgM was shown to play a role in intestinal [21], skeletal muscle [22], renal [23] and cardiac [24] models of IRI; it was hypothesised that the IgM in this context was critical for activating complement via the lectin pathway [25].

The role IgM and B cells play in acute liver injury is largely unknown and it was proposed that IgM and/or B cells would play a significant role in its pathogenesis.

## Methods

### Ethical approval & Welfare

#### Animal

Following local ethical approval at the University of Edinburgh, animal work was carried out according to UK Home Office regulations (Animals Scientific Procedures Act 1986) under licenses 60/4045 and 60/2605. Mice were housed under specific pathogen-free conditions at the University of Edinburgh. General anaesthesia (GA) was induced via inhaled isoflurane and post-operatively subcutaneous opioid analgesia (buprenorphine) was administered. Animals were sacrificed under GA by terminal exsanguination by way of cardiac puncture.

#### Human

The Tissue Governance Unit (South East Scotland SAHSC BioResource) provided ethical approval for the human tissue used in this study.

### Animals

All wild type (WT), knockout and transgenic mice were on a C57BL/6J background and matched for age and sex. RAG1-/- lack IgM and both mature B and T cells [26]. μMT mice lack mature B cells and IgM [27]. CD3εKO mice lack mature T cells [28].

### Models of liver injury

In this model of warm partial hepatic IRI, an atraumatic clamp was applied to the vascular pedicle supplying the left lobe for 40 minutes; the liver was then allowed to reperfuse for between 1 hour and 7 days. Care was taken to maintain intraoperative core body temperature at 36°C to minimise the masking effects of hypothermia on liver IRI [29]. In serum reconstitution experiments, 500μl serum from either wildtype or RAG1-/- donors was injected i.p. 24 hours pre-operatively; successful engraftment was confirmed by the deposition of IgM within areas of liver injury.

Paracetamol-induced liver injury and concanavalin A (con A) hepatitis models were used as alternative models of sterile liver inflammation. Briefly, following a period of 18–24 hours of fasting, mice were given a 200mg/kg intraperitoneal injection of paracetamol (acetaminophen, Sigma-Aldrich, Poole, UK). Con A hepatitis is a model of immune-mediated hepatitis [30] and was induced by an intravenous injection of 10mg/kg con A (Sigma-Aldrich). In both models, the injury was assessed at 24 hours post-injection.

The extent of any liver injury was assessed in terms of the serum level of alanine aminotransfersase (ALT), a biochemical marker of liver injury [31]. ALT was measured on a Cobas Fara centrifugal analyser (Roche Diagnostics Ltd, Welwyn Garden City, UK) using a commercial kit (Alpha Laboratories Ltd, Eastleigh, UK). ALT was correlated with the histological evidence of injury seen on sections stained with haematoxylin and eosin (H&E).

### Immunohistochemistry

The Tissue Governance Unit (South East Scotland SAHSC BioResource) kindly provided formalin-fixed sections from the explanted livers of patients requiring transplantation following paracetamol overdose; tissue blocks were specifically chosen by a consultant pathologist with a special interest in transplantation to include areas of both injured and uninjured liver. Antigen retrieval on these sections was performed by heating samples with Borg Decloaker [Biocare Medical, Birmingham, UK]. Tissue from the models of murine liver injury were fixed in methacarn and did not require a specific stage of antigen retrieval.

Sections were deparaffinised and rehydrated before endogenous peroxidase and avidin/biotin activity were quenched, prior to incubating with a goat anti-mouse IgM antibody (AI-2020, Vector Laboratories, Peterborough, UK) at a dilution of 1 in 5000 or goat anti-human IgM antibody (AI-3020, Vector Laboratories) at a dilution of 1 in 1000. Slides were subsequently incubated at room temperature with a polyclonal rabbit anti-goat biotinylated secondary antibody (E0466, DAKO, Ely, UK) at 1 in 400 dilution for 40 minutes. Sections were then developed with VectaStain RTU Elite (Vector Laboratories) followed by diaminobenzidine, before being counterstained.

IgM deposition was quantified from 6 randomly taken images at 200x magnification from each sample using ImageJ software (NIH, Bethesda, USA); it is expressed as percentage of total area.

### Flow cytometry

Single cell preparations from the liver were generated by a combination of mechanical (GentleMACS, Miltenyi Biotec, Bisley, UK) and enzymatic (2mg/ml Collagenase D, Roche) digestion. This was then passed through a 70μm filter and centrifuged at 50g for 5 minutes to remove hepatocytes, clumps of cells and debris. Red cells were lysed (Red Cell Lysis Buffer, Sigma-Aldrich). Immune cells were isolated by positive selection using a CD45+ MicroBead AutoMACS separation (Miltenyi Biotec) and then stained with a fixable Live-Dead marker (Life Technologies, Paisley, UK) and a multi-colour panel of antibodies, including CD3, CD4, CD8, CD19 and NK1.1 (Biolegend, San Diego, USA). Samples were then run on a BD LSR II Fortessa (BD Biosciences, Oxford, UK) and analysed with FlowJo software (Tree Star, Ashland, USA). B cells were defined as CD3-CD19+ cells, within a forward-side scatter defined lymphocyte gate.

### Statistical analysis

Groups were analysed with the aid of Prism 5 for Mac OSX [Graphpad Software, La Jolla, USA]; statistical methods are referred to specifically in the results section.

## Results

### IgM is deposited within areas of injured human and murine liver

**Fig 1:**
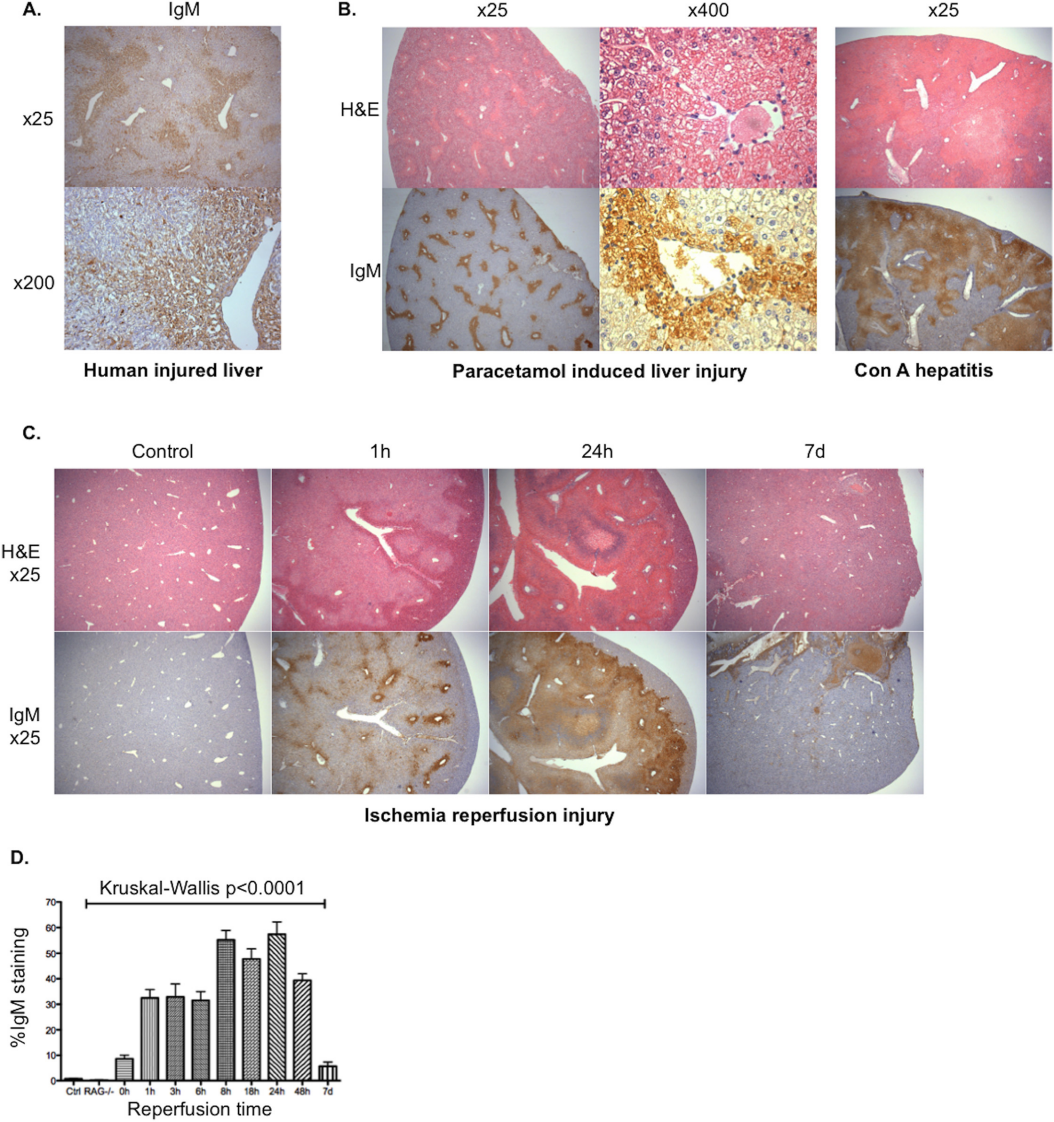
IgM deposition in acute sterile liver injury. Tissue sections of explanted livers from patients transplanted for fulminant liver failure secondary to paracetamol overdose were stained looking for evidence of IgM deposition; extensive deposition of IgM is demonstrated within areas of parenchymal injury (**A**). Tissue sections from male WT mice, with demonstrable sterile liver injury on sections stained with H&E following injection of 200mg/kg paracetamol or 10mg/kg con A also showed extensive IgM staining upon immunohistochemical staining (**B**). Male mice underwent 40 minutes left lobe ischemia and were then reperfused for 0 hours to 7 days (n = 2–6 per timepoint). The extent of IgM deposition was compared with the histological injury seen on slides stained with H&E; x25 magnification slides are shown at selected time points (**C**). IgM deposition was calculated as a percentage of total section area across the time-course (median + Standard Error of Mean (SEM)) (**D**); this deposition of IgM was rapid (seen within 1 hour) and disappeared with macroscopic resolution of injury.

To investigate whether IgM was deposited in areas of acute liver injury, sections from the explanted livers of patients transplanted for fulminant liver failure following paracetamol overdose were stained for IgM. Significant staining for IgM was seen in areas of injury (Fig 1A). This deposition of IgM was also found in 3 murine models of acute liver injury (Fig 1B & 1C): paracetamol induced liver injury, con A hepatitis and hepatic IRI. All 3 models demonstrated a similar zone 3 pattern of IgM deposition (and injury) to that seen in the explanted human liver. IgM was deposited rapidly in areas of liver injury and was found to disappear with macroscopic resolution of injury (defined as restoration of normal liver architecture) (Fig 1C & 1D).

### IgM is not critical to the pathogenesis of injury

**Fig 2:**
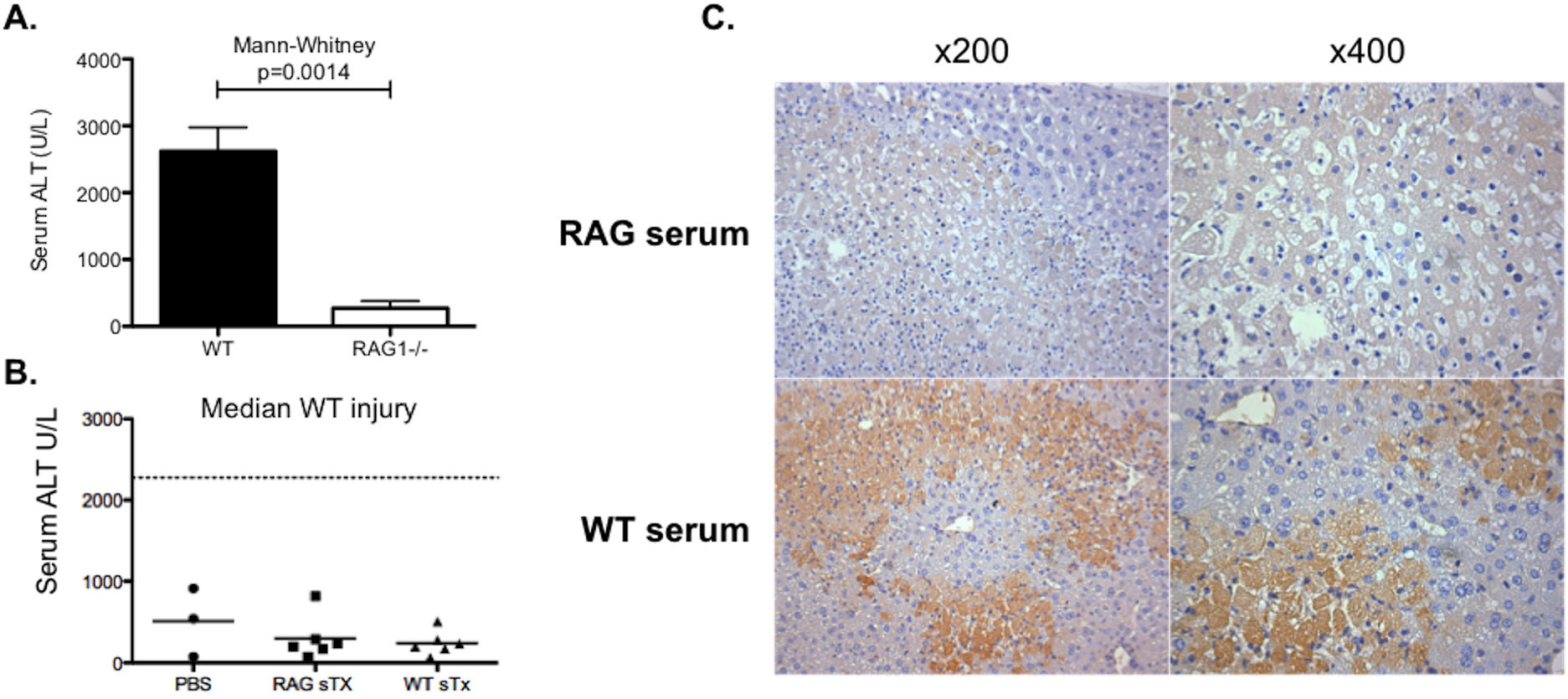
The pathogenicity of IgM in IRI. WT and RAG1-/- (n = 6) mice underwent experimental warm partial hepatic IRI (40 minutes ischemia and 24 hours reperfusion) to elicit the role T cells, B cells and/or IgM play in the pathogenesis of injury. There was significant reduction in the serum biochemical marker of injury (ALT) observed in RAG1-/- mice compared to WT controls (Mann-Whitney p = 0.0014, median + SEM) (**A**). To assess whether this protection was a result in the deficiency of IgM, RAG1-/- were injected with serum (sTx) from WT (n = 6) or RAG1-/- (n = 6) donors 24 hours prior to IRI. RAG1-/- mice were injected with PBS (n = 3) as an additional control group. Following 40 minutes ischemia and 24 hours reperfusion, there was no reconstitution of injury (median WT injury calculated from n = 32 mice) or significant differences between the groups [Kruskal-Wallis p = 0.66] (**B**). Successful engraftment of IgM from WT donors was confirmed by the deposition of IgM into areas of injury (**C**).

RAG1-/- are deficient in mature T cells, B cells and IgM [26]. RAG1-/- mice, which lack IgM and both mature B and T cells, were significantly protected from IRI (Fig 2A). In order to assess the importance of IgM in the pathogenesis of acute liver injury, serum from WT mice was transferred into RAG1-/- recipient mice. This failed to significantly reconstitute injury (Fig 2B), despite successful engraftment of IgM as evidenced by the deposition of IgM within visible areas of liver injury (Fig 2C). In spite of rapid IgM deposition within areas of liver injury (Fig 1), IgM does not appear to be critical to the development of significant ALI.

### B cells are not critical to injury

Given the protection seen in RAG1-/- mice was not attributable to deficiency in immunoglobulins normally present in serum (Fig 2), it was decided to investigate whether B cells themselves were a major contributor to injury.

**Fig 3:**
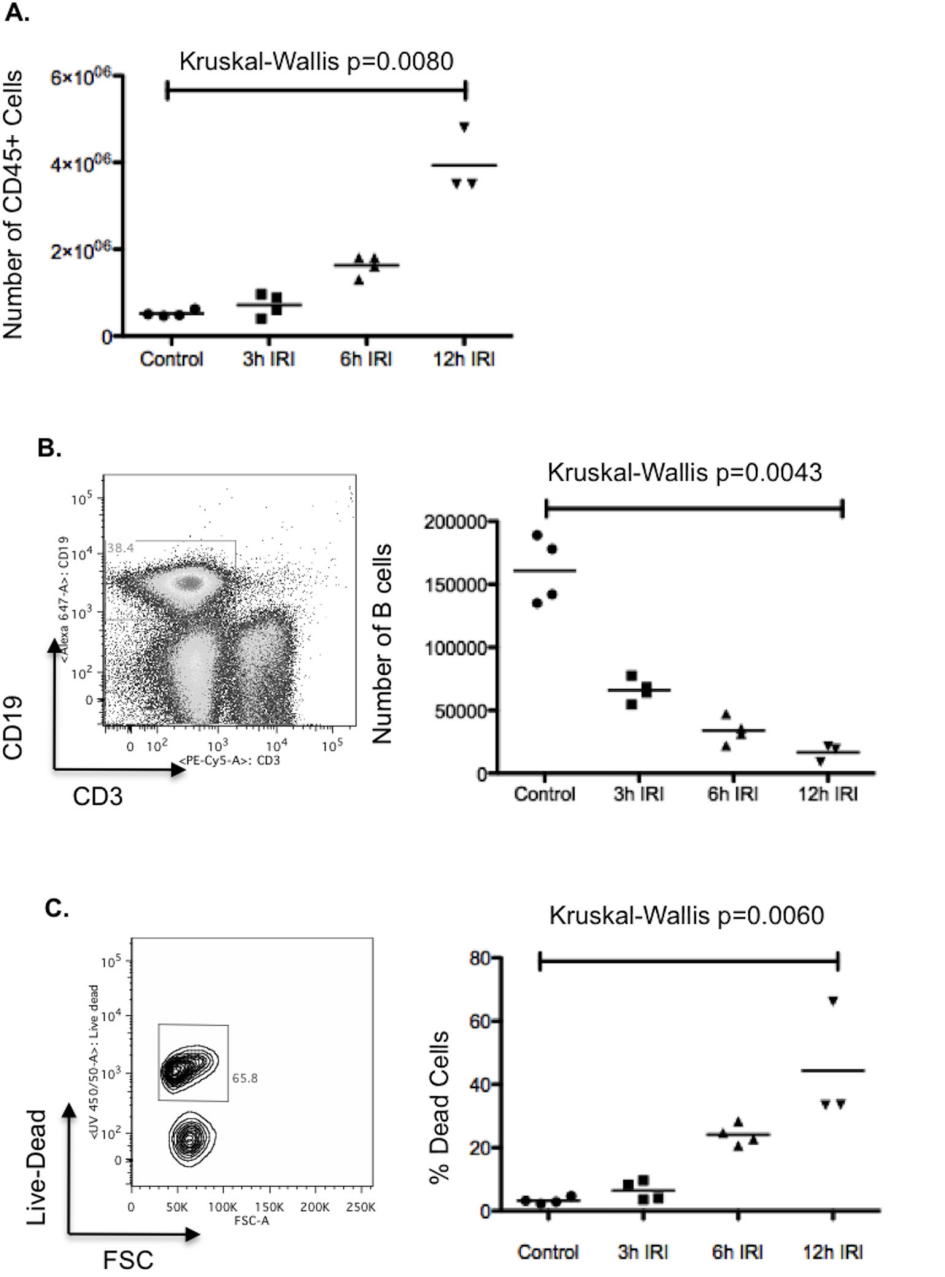
B cells following hepatic IRI. Following 40 minutes left lobe ischemia, WT mice were allowed to reperfuse for 3, 6 or 12 hours. Mice were then sacrificed and their ischemic left lobes digested. The isolated immune cells were analysed by flow cytometry. There was a significant influx of immune cells (defined as CD45+ cells) into the injured left lobe [Kruskall- Wallis p = 0.0080] (**A**). B cells were defined as CD3-CD19+ gated lymphocytes. The number of live B cells was found to decrease significantly with time following reperfusion [Kruskall- Wallis p = 0.0043] (**B**); this was predominantly due to cell death (as defined by positive staining for a live-dead marker) [Kruskall- Wallis p = 0.0060] (**C**).

Following reperfusion, there was a rapid accumulation of immune cells within the injured liver (Fig 3A); these were predominantly neutrophils (data not shown). This rapid influx of neutrophils fits with seen by other authors in the field [32]. Despite the increase in the number of immune cells, there was a progressive decrease in the number of live B cells following reperfusion (Fig 3B); this decrease is due to cell death (rather than an efflux) of B cells (Fig 3C).

**Fig 4:**
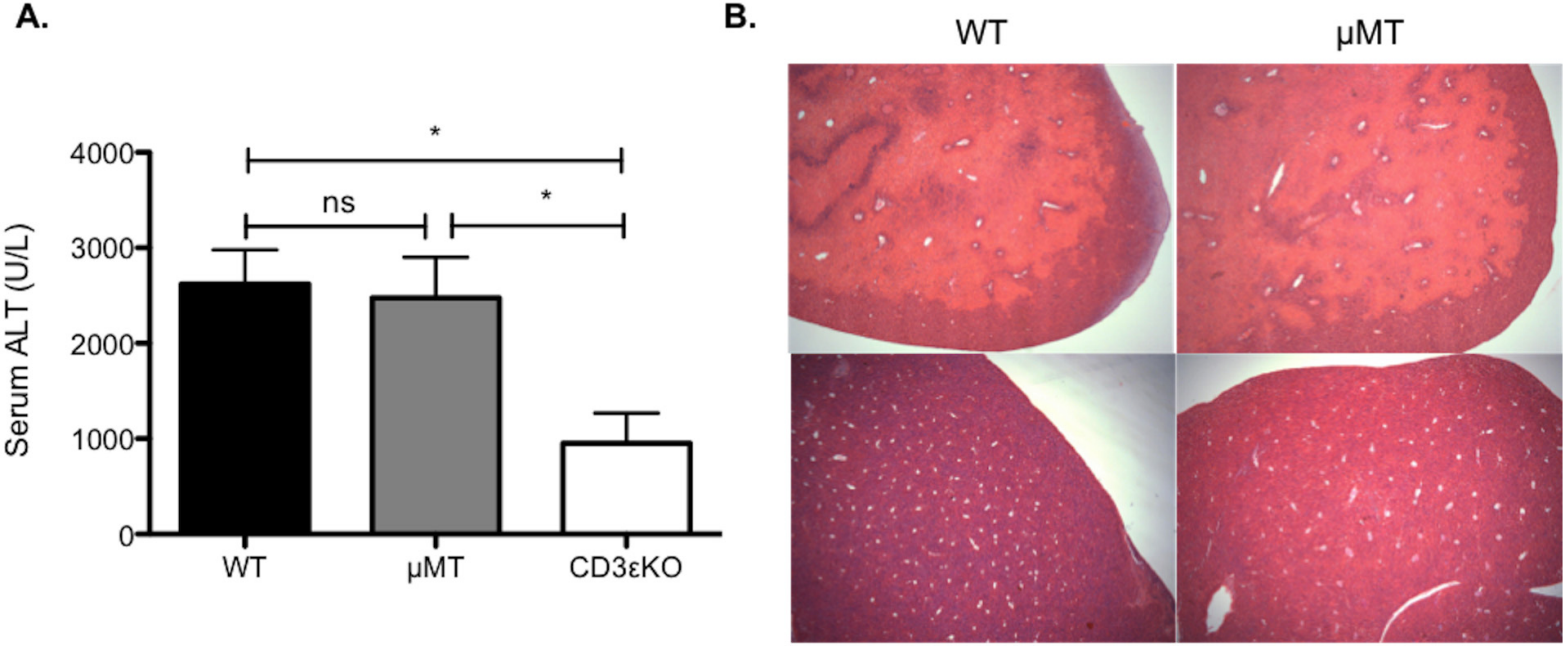
The role of B and T cells in hepatic IRI. To investigate whether the protection seen in RAG1-/- mice resulted from B and/or T cells, WT controls and mice deficient respectively in mature B cells (μMT) and T cells (CD3εKO) underwent 40 minutes left hepatic lobe ischemia (n = 12/group). Following 24 hours, reperfusion a significant reduction (Kruskall-Wallis p = 0.0037) was seen in mice lacking T, but not B cells.

B cell deficient (μMT) mice were subjected to hepatic IRI and no significant protection was seen compared to wild type controls (Fig 4). This is further evidence that IgM is not critical to injury, as these mice also lack IgM, yet still get significant injury. In contrast mice deficient in T cells (CD3εKO) mice, were significantly protected from injury (Fig 4). This points to the T cell compartment being a critical mediator of injury and explains the protection seen in RAG1-/- mice, given the apparent lack of pathogenicity of IgM or B cells in this setting.

### B cells and IgM are not critical to the resolution of injury

**Fig 5:**
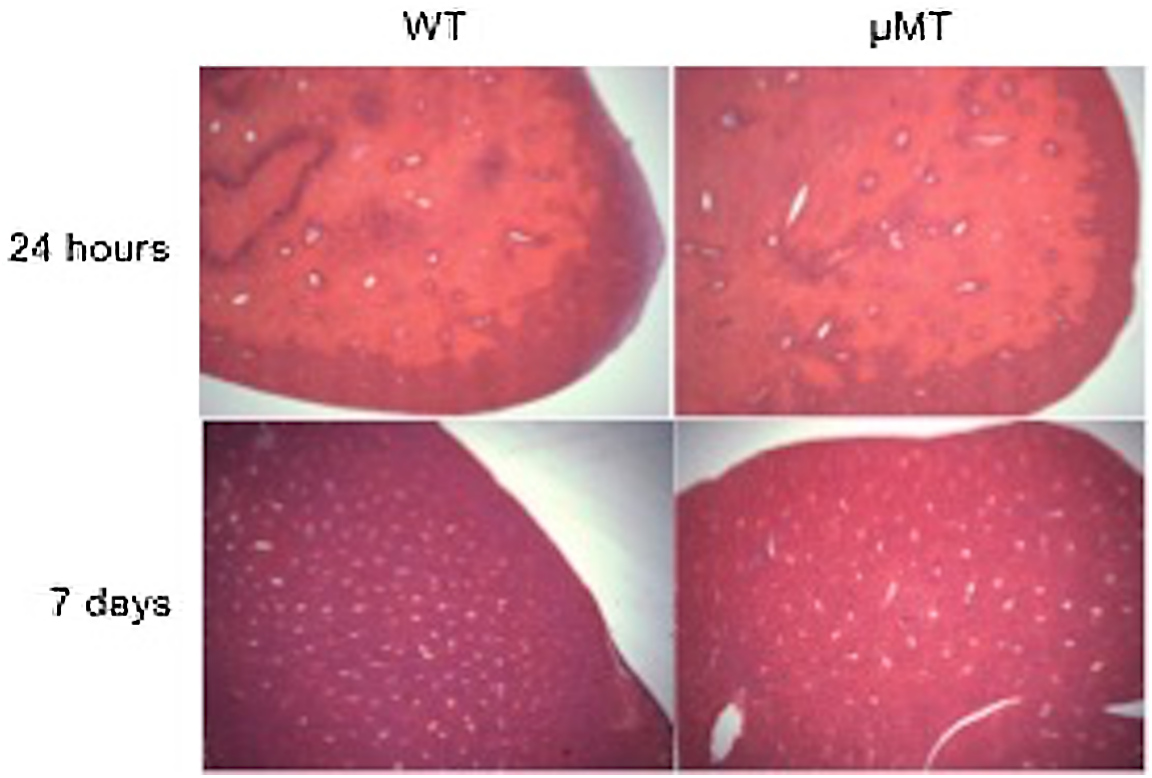
IgM and B cells are not critical to resolution of injury in the liver. Given the apparent lack of pathogenicity of IgM or B cells in this model of hepatic IRI, it was decided to see if they had a significant role in the resolution from injury. The recovery from injury was compared between WT and μMT mice. Representative H&E sections (x25 magnification), demonstrate similar injury at 24 hours, which resolved almost completely by day 7 post-IRI in both WT and μMT mice (residual necrosis<2% all mice [WT (n = 3), μMT (n = 4)]).

Deposition of IgM occurred early after injury and disappeared with injury resolution (Fig 1). To test the hypothesis that IgM and/or B cells could play an important role in the resolution of acute liver injury (e.g. by activating complement and promoting the opsonisation of cells for subsequent phagocytic clearance), mice underwent IRI and the livers were examined at 7 days post-injury to see if there was a significant delay in injury resolution in the absence of IgM and B cells. There was no significant delay in repair in μMT compared with wild type mice (Fig 5), with all mice at 7 days in both groups having <2% histological evidence of residual injury.

This implies that neither B cells nor IgM were important in the resolution of tissue injury in the post-ischemic liver.

## Discussion

Paracetomol toxicity and hypoxic liver injury typically cause zone 3 necrosis. In this study, the pattern of IgM staining in experimental models of these injuries, as well as in sections of liver from patients transplanted as a result of liver failure induced by paracetomol toxicity, mirrored this zone 3 distribution of injury (Fig 1). Deposition of IgM appears to be a good marker of the extent of liver injury, mapping closely the hepatocellular injury identified in serial H&E sections. Based on work with mouse strains deficient in IgM, including serum transfer experiments, this appears to be an epiphenomenon or at least that there is sufficient redundancy in the mechanisms underpinning sterile liver injury that IgM does not have a critical role in either the pathogenesis or subsequent resolution of acute liver injury (Figs 2 & 5). In other organ systems, IgM may have a more significant pathogenic role in the aetiology of IRI [21–24]. Although it should be noted that Lobo et al published conflicting data in which they found significantly worse renal IRI (and increased cardiac allograft rejection) in mice lacking IgM (IgMko), pointing to some of the important anti-inflammatory properties of IgM including inhibition of TLR-4–induced NF-κB nuclear translocation [33].

Given the time-frame in which this occurs (hours), the IgM “painting” injured cells is likely to represent polyreactive natural antibody (IgM) produced by B cells and found naturally in serum [20]. This “painting” of cells with IgM, has been described in other examples of tissue stress [34] and has been found to optimise complement mediated phagocytosis of apoptotic cells [35, 36]. Ciurana and colleagues found that serum IgM was essential for activating complement by necrotic cells [37]. The exact nature of the molecular target bound by IgM in this context remains unclear, but may result from the binding of natural antibody to “hidden” DAMPs exposed as a result of the sterile injury.

We also found that RAG1-/- mice, which also lack both B and T cells, were significantly protected from injury (Fig 2A); this correlates with work from other groups who observed similar degrees of hepatoprotection in these mice [16, 17]. We found that mice deficient in B cells (μMT), but with a functional T cell compartment, were not protected from injury and had no delay in their recovery (Figs 4 & 5). The role of B cells has not previously been reported in hepatic IRI. In work on renal IRI published by Burne-Taney and colleagues, they saw significant protection in the same strain of B cell deficient mice [18]. However, they were unable to recapitulate injury with adoptive transfer of B cells prior to injury, but did partially recreate injury by a serum transfer. A subsequent paper from the same group, demonstrated that the presence of B cells was associated with delayed repair in the post-ischemic kidney [19]. Others have not been able to reproduce this protection and conversely found that μMT mice sustained worse renal IRI compared to controls and postulated that this was due to the decreased levels of IL-10 they observed in these mice [23].

This is the first study, to look directly at the role T cells play in hepatic IRI with the use of a specific T cell knockout mice (CD3εKO). Work using TCR-/- mice has been performed in models of renal IRI, where T cells were also found to be key mediators of injury [38, 39]. The protection seen in T cell deficient (CD3εKO) mice, but not B cells and IgM deficient (μMT) mice, points to the protected phenotype seen in RAG1-/- mice by us and others [16, 17] arising from the T cell compartment, rather than from B cells or immunoglobulins (Fig 4). Data here adds directly to that from other labs, whose work has previously suggested a pathogenic role for T cells in hepatic IRI [40–42]. Conflictingly, Caldwell et al found significantly worse injury in CD4-/- mice, which could be attenuated with the adoptive transfer of CD4+ lymphocytes [43]. We have previously shown that (CD4+Foxp3+) regulatory T cells do not play a critical role in injury [44]. T cells in IRI have been variously found to be both antigen-dependent [41] and antigen independent [40]. CD8+ T cells have not been implicated in the aetiology of this injury across a number of models of IRI [10, 32]. While data from some groups suggest it is conventional (CD4+) T cells that are the key mediators of hepatic IRI [43, 45], others point to non-conventional (NKT, γδ) T cell populations [41, 42]. Further studies are needed to more fully understand the role T cells play in this injury.

Based on our data, therapeutic intervention targeting IgM or B cells (such as with Rituximab) would have no significant benefit to patients or impact on their recovery following liver injury.
